# Prescriptions

**Published:** 1895-01

**Authors:** 


					﻿FOR ECZEMA.
B. Salicylic acid .......... 3	j.
Zinc oxide.............. 3	iij.
Powdered starch......... 3	iv-
Wool-fat cerate......... 5	j.
Make ointment and apply.
—The Practitioner (London).
FOR REMOVAL OF FRECKLES.
B. White precipitate....... ) z-
Subnitrate of bismuth .. ( aa 3J*
Glycerin ointment......J ss.
M. Apply every other day.
—American Doctor.
GRANULAR CONJUNCTIVITIS.
La Reforma Med. has this pre-
scription :
B. Hydrarg. oxidi flavi ... .gr. iii.
Zinci Oxidi...............
Thymoli...............
Oocainae hydrochlor.. aa gr. iss.
Camphorae. ...........gr. ss.
Ungt. petrolati.......3 vi.
M. Sig.—Apply locally.
By J. C. Falk, M. D., Ph.D.,
St. Louis.
TO PREVENT IODI8M.
The following method of exhibit-
ing iodide of potassium is said to be
effectual in preventing the unto-
ward effects of the drug, as coryza,
depression, etc.:
B. Potassi iodidi....... J ss.
Ferri.etammon,citratis 3 j.
Tr. nucis vomicae...... 3 ij.
Aquae................. §	jss.
Tr. cinchon. comp. .ad. 3 iv.
M. Sig—Give a teaspoonful in
water after each meal.
RESORCIN DANDRUFF WASH.
B. Resorcin.
Ether.
Olive oil..............aa	3 i.
Alcohol ............... 5	vi.
Shake well and apply to the
scalp on a bristle brush twice as
large as the ordinary mucilage
brush, by insinuating it between
the locks of hair. The head should
be well washed with soap and warm
water twice a week.—Therapy.
Prescription Department.
TON8ILITI8.
B. 01. eucalyptus glob ... ,m xv.
Spir. camphor..............3 iss.
Tinct. guaiac.........3 iiiss.
Glycerin.......q. s. ad. § j.
Ten drops on sugar to dissolve in
the mouth every hour or two.
BRONCHIAL AND PULMONARY
TROUBLES.
The following formulae are said to
be of great service in all bronchial
and pulmonary troubles:
B. Terraline........ .......$	iv.
Creasoti (beechwood) ..3 ss.
M. S.—Take a teaspoonful every
four hours.
B. Terraline...............5 iij-
« yjn xerici ........... 3 ij.
M. S.—Take a teaspoonful every
|j t four hours.
GONORRHOEA.
Kava-kava, fluid extract, thirty to
sixty minims every four hours, con-
rols and effectually relieves gonor-
rhoea in from fifteen to thirty days.
—American Doctor.
dysmenorrhea.
Dr. W. Blair Stewart (Amer.
Therapist) claims to have had very
satisfactory results from the fol-
lowing :
B.	Cupri arsenitis...gr. 1-100.
Tinct. pulsatillae.gtt. vii.
Tinct. nucis vomicae.. gtt. iv.
Aquae................5 ii.
M. Sig.—Teaspoonful every half
hour or hour.
ABRASIONS—CUTANEOUS DISORDERS.
This antiseptio adhesive oint-
ment protects the surface of the
wound and is of special service in
dressing wounds of the face, and
valuable in cutaneous eruption,
excoriation and ulceration:
B. Zinci oxidi......grs.	v.
Zinci chloridi.. .grs.	xx.
Gelatinae...... 3vi.
Listerine.... 5 vii. M.
The gelatine to be dissolved in
the listerine by aid of gentle heat.
				

